# β-Glucans from *Trametes versicolor* (L.) Lloyd Is Effective for Prevention of Influenza Virus Infection

**DOI:** 10.3390/v14020237

**Published:** 2022-01-25

**Authors:** Shaohua Shi, Lei Yin, Xuehuai Shen, Yin Dai, Jieru Wang, Dongdong Yin, Danjun Zhang, Xiaocheng Pan

**Affiliations:** 1 Anhui Province Key Laboratory of Livestock and Poultry Product Safety Engineering, Livestock and Poultry Epidemic Diseases Research Center of Anhui Province, Institute of Animal Husbandry and Veterinary Science, Anhui Academy of Agricultural Sciences, Hefei 230031, China; sshua2013@163.com (S.S.); yinlei1989@yeah.net (L.Y.); xuehuaishen1986@126.com (X.S.); daiyin2020@163.com (Y.D.); wangjr0317@163.com (J.W.); yindd160@163.com (D.Y.); 2 CAS Key Laboratory of Pathogenic Microbiology and Immunology, Institute of Microbiology, Chinese Academy of Sciences, Beijing 100101, China

**Keywords:** *Coriolus versicolor*, β-glucans, influenza virus, prevention

## Abstract

*Coriolus versicolor* (*C. versicolor*) is a higher fungi or mushroom which is now known by its accepted scientific names as *Trametes versicolor* (L.) Lloyd. Many studies have shown that β-glucans from *C. versicolor* have various physiological activities, including activating macrophages to protect against *Salmonella* infection. However, whether β-glucans have antiviral effects has not been reported. Hence, the objective of this study was to confirm whether β-glucans could boost the immune response to combat influenza virus in mouse and chick models. The results show that β-glucans induced the expression of *Dectin-1*, costimulatory molecules (*CD80*/*86*) and cytokines *IL-6*, *IL-1β*, *IFN-β* and *IL-10* in murine bone marrow dendritic cells (BMDCs). In addition, orally administered β-glucans reduced weight loss, mortality and viral titers in the lungs of mice infected with influenza virus and attenuated pathological lung damage caused by the virus in the mice. Orally administered β-glucans improved survival and reduced lung viral titers in chickens infected with H9N2 avian influenza virus. These results suggest that β-glucans have a significant antiviral effect. Therefore, β-glucans could become a potential immunomodulator against influenza virus.

## 1. Introduction

Avian influenza (AI) is a highly contagious infectious disease of birds caused by influenza A virus, which is classified as a class A infectious disease by the International Veterinary Office. Although H9N2 subtype avian influenza virus (AIV) is a low-pathogenic AI, it is widely prevalent worldwide and is one of the major pathogens affecting poultry in Asia. The H9N2 subtype AIV not only carries some or all of the internal genes of the H7N9, H6N1, H10N8 and H5N6 subtypes but can also directly infect people [1]. These cases point to the urgent need to control the H9N2 virus. In addition, a recent study found that novel reassortant H1N1 subtype AIV was detected from wild bird feces [2], which can co-circulate among wild birds, pigs, and human beings [3]. Since AIV is prone to genetic mutation and rearrangement, the existing vaccine strains are no longer effective in protecting against infection from circulating strains. Therefore, there is an urgent need to develop a safe, effective, inexpensive and easily available oral drug for the prevention and control of AIV infection.

Medicinal fungi have attracted extensive attention due to their various pharmacological activities and biological functions. *Trametes versicolor* (L.) Lloyd (*Coriolus versicolor*, *C. versicolor*) is a traditional medicinal fungus that was reported in the Compendium of Materia Medica years ago in China [4]. *C. versicolor* is also a worldwide medicinal fungus. Many studies have shown that the polysaccharide of *C. versicolor* can activate or enhance the phagocytosis of reticulo endotheliocytes and macrophages, activate T lymphocytes and B lymphocytes, activate complement, promote the production of interferon and interleukin, and play various regulatory roles in the immune system [5,6,7]. Our previous study showed that the β-glucan of *C. versicolor* does not have any toxicity or side effects on cells [8]. Many studies have found that β-glucan extracted from yeast and fungi can play an antiviral role. The administration of 1,3-1,6 β-glucans to deformed wing virus (DWV)-infected honeybees reduces the DWV viral load compared with the control group [9]. Regla M showed that the β-glucan zymosan from yeast plays an important role in improving cytokine expression in vitro and in vivo, which enhances the immune response to spring viremia of carp virus (SVCV) infection [10]. Sulfated glucan from *Saccharomyces cerevisiae* has been shown to exhibit a remarkable adjuvant effect in the H5N1 vaccine in an animal model [11]. Previous studies have shown that β-glucan extracted from *Aureobasidium pullulans* provides protection against influenza virus [12]. β-glucans derived from *Lignosus rhinocerotis* (Cooke) Ryvarden, *Pleurotus giganteus* (Berk.) Karunarathnas and K.D.Hyde, *Hericium erinaceus* (Bull) Persoon, and *Schizophyllum commune* (Fr.) were the least toxic to Vero cells and provided significant protection against DENV2. Recent studies have shown that β-glucans derived from the edible shiitake mushroom *Lentinus edodes* may have potential therapeutic value for the cytokine storm caused by COVID-19 [13]. Hence, β-glucan extracts from mushrooms may be a potential substance against viral infection.

In this study, we confirmed the effects of β-glucans on the expression of costimulatory factors, dendritic cell-associated C-type lectin-1 (*Dectin-1*) and cytokine genes in murine bone marrow dendritic cells (BMDCs). Subsequently, we further observed the protective effect of β-glucans on mice and chicks infected with influenza virus or AIV.

## 2. Materials and Methods

### 2.1. Viruses, C. versicolor Extracts

The A/Puerto Rico/8/34 (PR8; H1N1) strain of influenza A virus and A/duck/Xuzhou/07/2003 (H9N2) virus strains were propagated in 9-day-old embryonated chicken eggs and were used to infect mice and chicks, respectively. The β-glucans from *C. versicolor* were isolated using hot-water extraction, which were processed according to previously published methods [8]. Briefly, the isolation and purification of the water-soluble polysaccharide from the *C. versicolor* mushrooms was accomplished by hot water extraction, filtration, solvent precipitation and dialysis. Using anion-exchange chromatography, the polysaccharides were separated from the crude polysaccharides using a DEAE-Sephacel column and a linear gradient of NaCl (0–2 mol/L). The molecular weight was around 750 kDa after Sepharose CL-4B gel filtration chromatography. The content of the β-glucan (β-(1→3, 1→4)-glycosidic bonds) extract from *C. versicolor* was detected enzymatically using a Megazyme kit (Bray, Ireland) and 86.5% of the dry weight was acquired.

### 2.2. Animals and Ethics Statement

Specific pathogen-free (SPF) female C57BL/6 mice, aged 68 weeks, were obtained from Vital River Laboratory Animal Technology Co., Ltd., Beijing, China. SPF whole leghorn layer chickens and SPF embryonated chicken eggs were purchased from Merial Vital Laboratory Aniamal Technology Co., Ltd., Beijing, China. All animal experiments were performed according to the procedures approved by the Anhui Academy of Agricultural Sciences and complied with all relevant ethical regulations regarding animal research. All surgeries were performed under anesthesia to minimize suffering.

### 2.3. Induction of BMDCs

BMDCs were isolated from the femurs and tibias of C57BL/6 male mice as previously published [14]. Briefly, the muscle tissue around femurs and tibias of the killed mice was removed, the bone marrow suspension was collected and centrifuged at 12,000× *rpm* for 5 min, and the supernatant was discarded. The cells were resuspended in red blood cell lysis buffer (BD, Franklin Lakes, NJ, USA), incubated at room temperature for 3–5 min, centrifuged at 12,000× *rpm* for 5 min, and washed twice with RPMI 1640 medium (Gibco, Grand Island, NY, USA) with 10% FBS. The cells were added to a 6-well culture plate (2 × 10^6^ cells/well), and then interleukin (IL-4) (1000 U/mL) (PeproTech, Cranbury, NJ, USA) and granulocyte-macrophage colony stimulating factor (GM-CSF) (1000 U/mL) (PeproTech, Cranbury, NJ, USA) were added. Every 2 days, the culture plate was gently shaken and 3/4 of the culture medium volume was replaced with fresh culture medium containing IL-4 and GM-CSF. On the eighth day, the cells were collected for subsequent experiments.

### 2.4. Real-Time Quantitative RT-PCR

Murine BMDCs were seeded into 24 plates (4 × 10^5^ per/well) and incubated with final concentrations of 200 µg/mL and 400 µg/mL β-glucans at 37 °C in 5% CO_2_ for 6 h. To assess gene expression in the murine BMDCs treated with β-glucans, real-time fluorescence RT-PCR was used to detect the copy number of the infected virus in the different treatments using the ABI 7500 real-time PCR platform. [8]. Total RNA was extracted from murine BMDCs using the MiniBEST viral RNA/DNA Extraction Kit Ver.5.0 (Takara, Beijing, China). The total RNA concentration was determined using a BioTek Epoch 2 microplate reader (BioTek, Winooski, Vermont, USA). The extracted RNA was reverse transcribed using a PrimeScript™ RT kit with gDNA Eraser (Takara, Beijing, China) following the manufacturer’s instructions. To detect the expression of all genes (*Dectin-1*, *CD80*, *CD86*, *IL-1β*, *IL-6*, *IL-10* and *IFN-β*), real-time RT-PCR in triplicate was carried out using SYBR Green PCR Master Mix for 40 cycles. All primers used in this study are listed in Table 1. To evaluate the results, all the values are provided as fold change by using the 2^−∆∆CT^ formula [8].

### 2.5. Virus Challenge in Mice and Chicks

Seventy-two C57BL/6 mice were divided into four groups with 18 mice in each group (PBS group, 200 µg/mouse group, 400 µg/mouse group and ribavirin group). Each mouse was intranasally (i.n.) challenged with 10 × lethal dose 50 (LD_50_) of the PR8 (H1N1) strain of influenza virus under anesthesia with mebubarbital (Avertin; Sigma) [15]. After infection, mice were orally gavaged with β-glucans (1 mg/mL, 0.2 mL/mouse and 2 mg/mL, 0.2 mL/mouse) or PBS once a day for 7 days. Ribavirin was given intramuscularly once a day for 7 days (66.6 mg/kg) as a positive control. All mice were weighed and observed for clinical symptoms for 2 weeks. The loss of weight (20%) was considered a humane endpoint, and the mice were humanely euthanized to meet this standard.

Sixty-twenty-day-old SPF white leghorn chicks were divided into four groups with 15 chicks in each group (PBS group, 200 µg/chick group, 400 µg/chick group and ribavirin group). All birds were i.n. infected with 10^6.0^ EID_50_ of H9N2 AIVs (A/duck/Xuzhou/07/2003). After infection, birds were orally gavaged with β-glucans (1 mg/mL, 0.2 mL/chick and 2 mg/mL, 0.2 mL/chick) or PBS once a day for 7 days. Ribavirin was given intramuscularly once a day for 7 days (66.6 mg/kg) as a positive control. Birds were monitored for two weeks by daily assessment for deaths.

Lung samples from the mice and chicks were collected on Day 5 postinfection (p.i.), and 10% (*w/v*) tissue homogenates were centrifuged and then assessed in 9-day-old embryonated chicken eggs according to published methods [16].

### 2.6. Histopathology

Pathological examination was performed according to published methods [17]. In short, lung samples from animals were taken and fixed in 4% paraformaldehyde. After dehydration, transparency, wax immersion and embedding, pathological sections with a thickness of 3 μm were made by an automatic paraffin embedding machine. Then, hematoxylin and eosin (H&E) staining was carried out, and histopathological changes were observed under a microscope.

### 2.7. Statistical Analysis

All data are expressed as the mean± SEM. GraphPad Prism 9.0 Software (San Diego, CA, USA) was employed to analyze data and draw bar graphs. All experiments were repeated at least three times independently. Univariate analysis of variance (ANOVA) and t-tests were used for comparisons between groups. *p* values < 0.05 were considered statistically significant.

## 3. Results

### 3.1. β-Glucans Induced the Expression of Dectin-1 and Costimulatory Factor Genes in BMDCs

**Figure 1:**
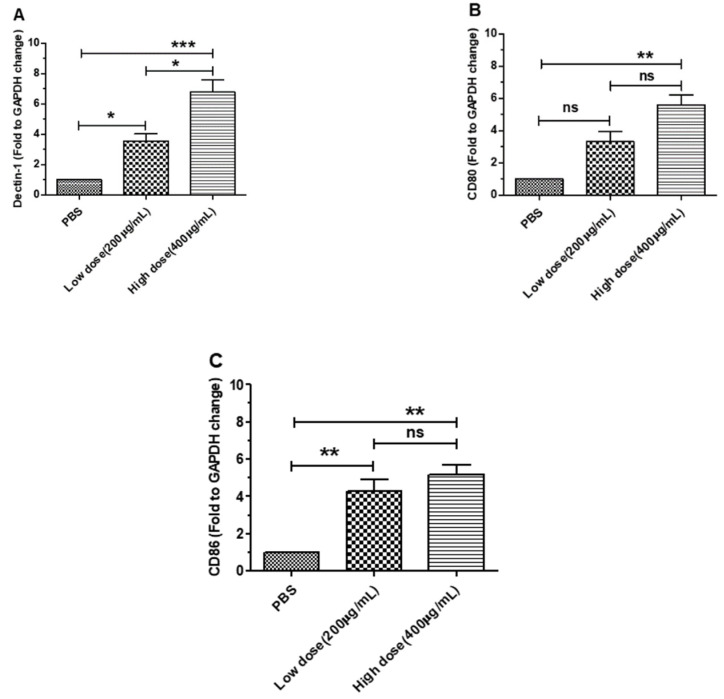
Effects of β-glucans on the expression of *Dectin-1* and costimulatory factors in BMDCs treated with β-glucans. Expression patterns of Dectin-1 (**A**) and costimulatory factors *CD80* (**B**) and *CD86* (**C**) in BMDCs at 6 h after treatment with 200 µg/mL and 400 µg/mL β-glucans. All data were expressed as the group mean± SEM. Significant differences between groups are indicated by asterisks (*). (* *p* < 0.05, ** *p* < 0.01, *** *p* < 0.001).

To test the effect on the relative expression level of Dectin-1 and costimulatory factors (*CD80* and *CD86*) in BMDCs, we cocultured BMDCs with different concentrations of β-glucans for 6 h. The results showed that *Dectin-1* was significantly expressed in BMDCs treated with β-glucans compared with the control group (*p* < 0.01) (Figure 1A). In addition, the results showed that the expression of *CD80* and *CD86* was significantly increased in BMDCs after treatment with β-glucans for 6 h (Figure 1B,C). These results suggest that β-glucans from *C. versicolor* can promote the expression of *Dectin-1* and costimulatory factors in BMDCs.

### 3.2. β-Glucans Promoted IL-1β, IL-6, IL-10 and IFN-β Gene Expression in BMDCs

**Figure 2:**
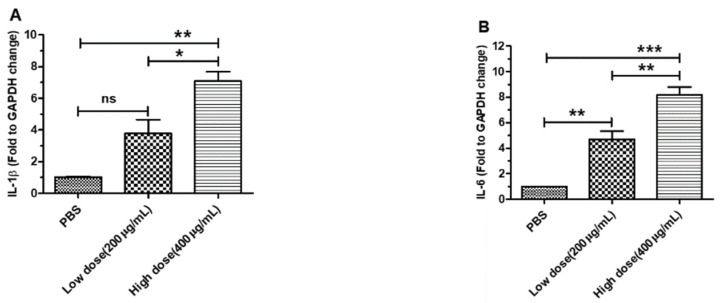

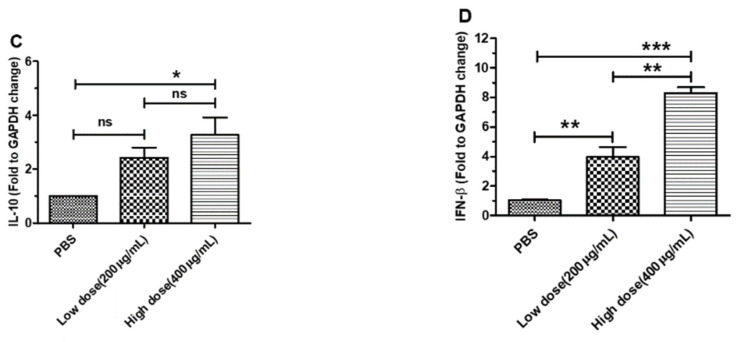
Effects of β-glucans on the expression of *IL-1β*, *IL-6*, *IL-10* and *IFN-β* in BMDCs treated with β-glucans. Expression patterns of *IL-1β* (**A**), *IL-6* (**B**), *IL-10* (**C**) and *IFN-β* (**D**) in BMDCs at 6 h after treatment with 200 µg/mL and 400 µg/mL β-glucans. All data were expressed as the group mean ± SEM. Significant differences between groups are indicated by asterisks (*). (* *p* < 0.05, ** *p* < 0.01, *** *p* < 0.001).

To analyze the role of the relative expression levels of the *IL-1β*, *IL-6*, *IL-10* and *IFN-β* genes in BMDCs, we cocultured BMDCs with different concentrations of β-glucans for 6 h. The results showed that the proinflammatory factors *IL-1β* and *IL-6* were significantly expressed in BMDCs treated with β-glucans compared with the control group (Figure 2A,B). However, our results suggested that β-glucans induced the expression of the anti-inflammatory cytokine *IL-10* in BMDCs (Figure 2C). Additionally, compared to that in the PBS group, the expression of *IFN-β* was dose-dependent in BMDCs treated with β-glucans (Figure 2D). Our results show that β-glucans from *C. versicolor* have different physiological functions in BMDCs.

### 3.3. β-Glucans Provided Protection against H1N1 Influenza Virus Infection in Mice

**Figure 3:**
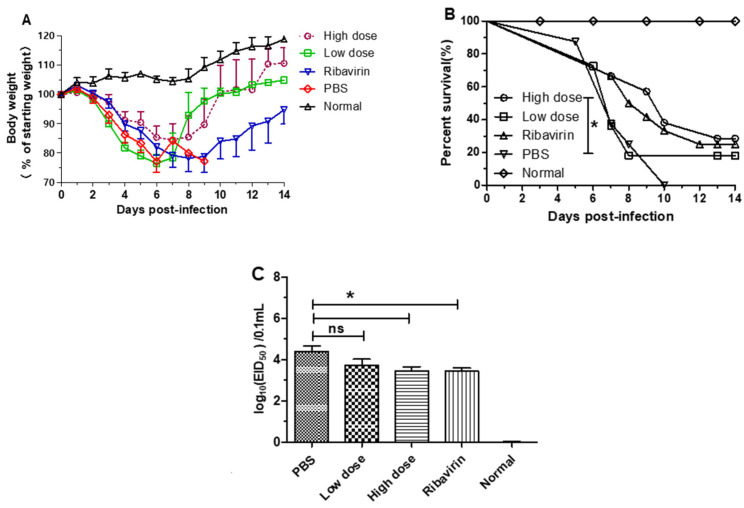
β-glucans provide protection against H1N1 influenza virus in mice. BALB/c mice were challenged with 10 × LD_50_ of A/PR/8/34 (H1N1) and then orally treated with a low dose (200 µg/mouse) and a high dose (400 µg/mouse) of β-glucans every day. (**A**) Weight loss (%) of mice was observed after infection with virus every 24 h (*n* = 10–13). (**B**) Percent survival of animals was monitored after challenge with H1N1 influenza virus (* *p* < 0.05) (*n* = 10–13). (**C**) Five days after challenge, the A/PR/8/34(H1N1) in the lungs was titered in chicken embryos. The data are shown as the means ± SEM of triplicate tests (*n* = 5) (* *p* < 0.05).

To evaluate the protective effect of β-glucans in mice, mice were first infected with 10 × LD_50_ of A/PR/8/34(H1N1) influenza virus and then orally administered different doses of β-glucans (Figure 3). The results showed that weight loss was significantly lower in the oral β-glucans groups (high dose and low dose) than in the PBS and ribavirin groups (Figure 3A). In addition, all mice in the saline group died after challenge with H1N1 influenza virus. However, the mortality of mice in the oral high dose group was significantly lower than that of the PBS group (Figure 3B). Five days after H1N1 influenza virus infection, the lungs of each group were titered on chicken embryos. Compared with the PBS group, there was a significantly lower viral titer in the high-dose and ribavirin groups (Figure 3C).

### 3.4. β-Glucans Alleviated the Lung Pathological Changes in Mice

**Figure 4:**
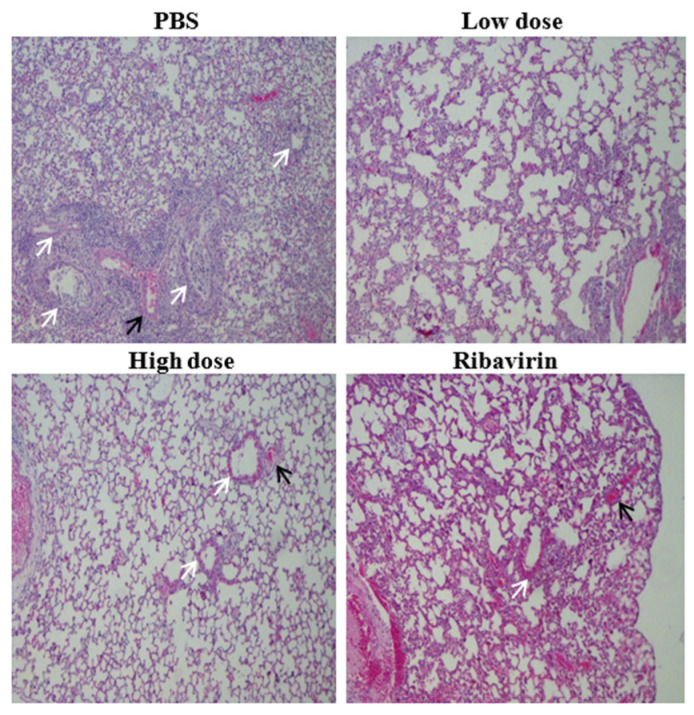
Pathological changes in the lungs of mice after challenge with A/PR/8/34(H1N1) (10 × LD_50_). Five days after infection, lungs were isolated from mice treated with low-dose (200 µg/mouse) and high-dose (400 µg/mouse) β-glucans, and the samples were observed after hematoxylin and eosin (H&E) staining. White arrows show a bronchus, and black arrows show a vessel.

To further evaluate the protective effect of β-glucans on mice, lung samples from each group of mice were observed for histomorphological examination after viral infection. Five days after viral infection, our results showed that severe interstitial pneumonia was found in the lungs of the PBS group, and there was marked infiltration of lymphocytes (Figure 4). However, histomorphological examination of the lungs showed that the symptoms of pulmonary interstitial pneumonia were less severe in the low-dose group than in the PBS group and that the symptoms of pulmonary interstitial pneumonia were less severe in the high-dose group than in the low-dose group (Figure 4). These results suggest that daily oral β-glucans can significantly reduce lung pathological damage in mice after viral infection.

### 3.5. β-Glucans Provided Protection against H9N2 AIV Infection in Chicks

**Figure 5:**
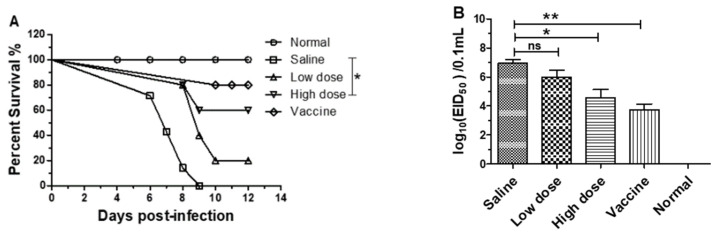
β-glucans provide protection against avian influenza virus in chicks. Birds were challenged with A/duck/Xuzhou/07/2003(H9N2) (1 × 10^6.0^ EID50) and then orally treated with a low dose (200 µg/chick) or a high dose (400 µg/chick) of β-glucans every day. (**A**) Percent survival of birds was monitored after challenge with avian influenza virus (* *p* < 0.05) (*n* = 10). (**B**) Five days after challenge, the A/duck/Xuzhou/07/2003(H9N2) in the lungs from birds was titered in chicken embryos. The data are shown as the means ± SEM of triplicate tests (*n* = 5) (* *p* < 0.05, ** *p* < 0.01).

To determine the protective effect of β-glucans on chicks, chicks were first infected with H9N2 subtype AIVs and then given different doses of β-glucans orally. As shown in Figure 5A, all the chicks in the PBS group died within nine days, and the survival rates for chicks in the low-dose group and in the high-dose group were 20% and 60%, respectively. Compared with the saline group chicks, there was a significantly higher survival rate in the high-dose group (Figure 5A). In addition, 5 days after the chicks were infected with virus, the lungs of chicks in each group were titered for virus. The results showed that the virus titer in the high-dose group was remarkably lower than that in the saline group (Figure 5B). Together, our results suggest that β-glucans are effective in the treatment of avian influenza virus infection in chicks.

## 4. Discussion

Dendritic cells (DCs) are the most functional professional antigen-presenting cells and can efficiently absorb, process and present antigens. DC-associated C-type lectin 1 (*Dectin-1*) can initiate and induce the innate immune response and plays a vital role in the differentiation, activation, and maturation of DCs [18]. *Dectin-1* can trigger DCs to drive T cell responses against several different fungal pathogens, bacteria, viruses and tumors [19,20]. In this study, our data showed that β-glucans promote the expression of *Dectin-1* in BMDCs, which may activate downstream signals to produce an immune response [21]. Mature DCs can express remarkable levels of the costimulatory molecules *CD80*/*86*, which promote T cell activation for immune function. Recent studies have reported that yeast-derived β-glucans cannot activate the expression of porcine BMDC surface costimulators [22]. However, we found that *C. versicolor*-derived β-glucans can activate the upregulation of *Dectin-1* expression in mouse BMDCs. One possible reason for this phenomenon is that BMDCs from different hosts lead to different sensitivities to β-glucans. Second, the physical properties of β-glucans may be different due to the different sources of sugar.

Previous studies have shown that particulate β-glucan activates TLR4 in human dendritic cells, resulting in increased production of the proinflammatory cytokines *IL-23*, *IL-4*, *IL-6*, and *TNF-α* [23]. Recent research has also found that β-glucan triggers porcine BMDCs to induce dose-dependent production of the pro- and anti-inflammatory cytokines *TNF-α*, *IL-1β* and *IL-10* [22]. In this study, our results showed that β-glucan treatment of mouse BMDCs promotes the expression of *IL-1β*, *IL-6*, *IL-10* and *IFN-β*. Consistent with previous studies, fungal β-glucans activate *IFN-β* secretion by DCs through the D receptor, and *IFN-β* promotes the expression of DC costimulators and secretes a variety of cytokines, including *IL-12 p70*, *IL-2*, *IL-6*, and *TNF-α* [24]. The mechanism of this phenomenon is the proliferation and activation of CD4 T cells or CD8 T cells and the production of *IFN-γ*, *IL-2*, and granzyme B, which may provide new ideas for the design of vaccines against pathogens and tumors [25].

There is much evidence that β-glucans increase survival in experimental animals after being challenged by pathogens [26,27]. In our previous work, we found that β-glucans induced the activation of macrophages, which enhanced the phagocytosis and clearance of *Salmonella* by producing nitric oxide, which provided effective protection against *Salmonella* infection in mice [8]. In addition, β-glucan from particulate yeast contributes to recruiting macrophages, DCs and effector T cells to the liver and increases the Th1-type immune response in HBV model mice, and β-glucans also play an important role in the elimination of HBV DNA [28]. In this study, oral β-glucans reduced weight loss and improved survival mortality in a model of mice infected with influenza virus. Our data further showed that β-glucans also significantly reduced viral titers in the lungs of mice. We found similar results that β-glucans could reduce mortality and virus titers in the lungs of chicks infected with H9N2 AIV. Studies have shown that (1,3)-β-D-glucan is involved in immune activation during human immunodeficiency virus infection [29]. β-glucan acts as an immune-stimulatory agent to increase cytokine expression and improve the response to spring viremia of carp virus (SVCV) challenge [10]. Conversely, sugar can reduce the inflammatory response and antioxidation in human cell lines, which can hopefully be used to develop a targeted drug against novel coronavirus [13]. These results suggest that different sources of β-glucan or structural differences may produce different physiological effects, but the mechanism remains to be further studied.

Many studies have shown that β-glucan plays an important role in inhibiting inflammatory damage [30,31,32]. The protective effect is closely related to the antioxidant and anti-inflammatory cytokines induced by β-glucan. Many studies have reported that β-glucan can significantly induce *IL-10* secretion, which plays an important function in inhibiting T cell activation and reducing histopathological damage. In this study, our data suggested that β-glucan induces *IL-10* expression and reduces virus-induced lung pathological inflammation in experimental animals. In addition, a recent study reported that β-glucan enhances NK cell killing activity by promoting *IL-10* secretion by porcine monocytes [33], which suggests that *IL-10* indirectly regulates NK killing function and improves antiviral effects. However, β-glucan inhibition of virus-infected lung injury may involve multiple mechanisms, and this protective mechanism will be revealed using gene deletion animal models in the future.

Many different sources of β-glucan can be used as vaccine adjuvants, such as avian influenza virus, Newcastle disease virus and infectious bursitis virus [11,34,35], which suggests that β-glucan is an excellent vaccine adjuvant and can significantly improve the host immune response. However, we found that oral administration of β-glucan also enhances the immune response against pathogen infection. The possible role is that oral β-glucan is different from other food substances; it is resistant to stomach acid and easily captured by DCs. Activated DCs can return to local lymph nodes (Peyer’s patches), inducing lymphocyte activation and differentiation, release of cytokines and systemic immune activation.

## 5. Conclusions

According to many previous studies, β-glucan has been reported to have multiple effects against infection. Our results confirm that β-glucan could induce BMDC activation and cytokine secretion in vitro. It also had a strong antiviral effect in experimental animals. This is a new strategy for using β-glucan as a potential antiviral drug or vaccine adjuvant against viral infections in the future.

## Figures and Tables

**Table 1:** Primer sequence of qPCR.

Gene Name	Genbank Accession No.	Forward Primer (5-3)	Reverse Primer (5-3)	Amplicon/bp
CD80	AF065894.1	CCCCAGAAGACCCTCCTGATAG	CCGAAGGTAAGGCTGTTGTTTG	172
CD86	NM_019388.3	GCCGTGCCCATTTACAAAGG	GTTCCTGTCAAAGCTCGTGC	160
Dectin-1	AY534909.1	TGGTAGTAGTGGTTGCTGCAGTGCTGGG	GTAGTTTGGGATGCCTTGGAGGGAGCCA	169
IL-10	NM_010548.2	GCCAGAGCCACATGCTCCTA	GATAAGGCTTGGCAACCCAAGTAA	145
IL-1β	NM_008361.4	GTTCCCATTAGACAACTGCACTACAG	GTCGTTGCTTGGTTCTCCTTGTA	162
IL-6	X54542.1	CGGCCTTCCCTACTTCACAAGTCCG	CAGGTCTGTTGGGAGTGGTATCC	142
IFN-β	X14455.1	CTGGCTTCCATCATGAACAA	CATTTCCGAATGTTCGTCCT	125
GAPDH	AA661116.1	CACTCACGGCAAATTCAACGGCAC	GACTCCACGACATACTCAGCAC	152

## Data Availability

Not applicable.
